# G-CSF and IL-8 for early diagnosis of sepsis in neonates and critically ill children – safety and cost effectiveness of a new laboratory prediction model: study protocol of a randomized controlled trial [ISRCTN91123847]

**DOI:** 10.1186/cc2971

**Published:** 2004-10-19

**Authors:** Thomas Horisberger, Stephan Harbarth, David Nadal, Oskar Baenziger, Joachim E Fischer

**Affiliations:** 1 Research Fellow, Department of Neonatology and Intensive Care, University Children's Hospital, Zurich, Switzerland; 2 Scientific Consultant, Infection Control Program, Geneva University Hospitals, Geneva, Switzerland; 3 Head, Division of Infectious Diseases, University Childrens's Hospital Zurich, Zurich, Switzerland; 4 Head, Department of Neonatology and Intensive Care, University Children's Hospital, Zurich, Switzerland; 5 Consultant, Department of Neonatology and Intensive Care, University Children's Hospital, Zurich, Switzerland

**Keywords:** children, cost effectiveness, prediction model, sepsis, study protocol

## Abstract

**Introduction:**

Bacterial infection represents a serious risk in neonates and critically ill paediatric patients. Current clinical practice is characterized by frequent antibiotic treatment despite low incidence of true infection. However, some patients escape early diagnosis and progress to septic shock. Many new markers, including cytokines, have been suggested to improve decision making, but the clinical efficacy of these techniques remains uncertain. Therefore, we will test the clinical efficacy of a previously validated diagnostic strategy to reduce antibiotic usage and nosocomial infection related morbidity.

**Methods:**

All patients admitted to the multidisciplinary neonatal and paediatric intensive care unit of a university children's hospital will be included. Patients will be allocated either to routine sepsis work up or to the intervention strategy with additional cytokine measurements. Physicians will be requested to estimate the pre-test probability of sepsis and pneumonia at initial suspicion. In the treatment arm, physicians will receive raw cytokine results, the likelihood ratio and the updated post-test probability. A high post-test probability will suggest that immediate initiation of antibiotic treatment is appropriate, whereas a low post-test probability will be supportive of watchful waiting or discontinuing prophylactic empirical therapy. Physicians may overrule the suggestions resulting from the post-test probability.

**Conclusion:**

This trial will ascertain the clinical efficacy of introducing new diagnostic strategies consisting of pre-test probability estimate, novel laboratory markers, and computer-generated post-test probability in infectious disease work up in critically ill newborns and children.

## Introduction

Bacterial infection is an important cause of mortality and morbidity in newborns and critically ill paediatric patients [1,2]. The high risks associated with untreated infection and the lack of accurate clinical or laboratory prediction methods result in a low threshold for initiating empirical antibiotic therapy. In neonatal and paediatric intensive care, antibiotic therapy is used in as many as 80% of patients, with an average of about 50% [3]. Only a minority of treated patients suffer from true infection. The majority receive antibiotics for 48–72 hours because clinical signs suggest possible infection and laboratory parameters are unable to rule out infection. In otherwise healthy newborns, this practice causes prolonged separation from the mother and increased the costs of care [4,5]. The high prevalence of unnecessary antibiotic therapy augments the risk for selecting resistant bacterial strains. Despite liberal antibiotic prescription, in some patients sepsis is not diagnosed until they have progressed to serious conditions such as septic shock.

Several groups have suggested that measurement of cytokines may be done to facilitate early diagnosis [6-8]. We previously reported diagnostic test accuracy studies in which we derived a prediction model based on the measurement of plasma levels of granulocyte colony-stimulating factor (G-CSF) and IL-8, and tracheal aspirate levels of G-CSF [9,10]. If plasma cytokine concentrations rise above pre-specified thresholds, then serious bacterial bloodstream infection is highly likely. Gram-negative sepsis is practically excluded if plasma levels remain low. Although plasma measurements assist in ruling out life-threatening sepsis, localized infections such as ventilator-associated pneumonia [11] cannot be diagnosed on the basis of blood derived cytokine concentrations. However, we previously showed tracheal aspirate levels of G-CSF to assist in diagnosing ventilator-associated pneumonia [10], which is the most frequent reason for prescribing antibiotics in our unit [3]. We recently conducted validation studies for plasma measurements of IL-8 and G-CSF and tracheal aspirate levels of G-CSF, employing a new laboratory method that allows simultaneous determination of parameters from 50 μl blood or tracheal aspirate. We refined the fluorescent bead-based immunoassay to reduce the assay turnaround time from 4.5 hours to 2 hours, rendering it suitable for routine clinical use.

To assess the clinical efficacy of the new diagnostic measures, we suggest that a randomized controlled trial be conducted comparing two management strategies. The control strategy will consist of routine management, with the exception that physicians are requested to provide a probability estimate for the presence of bacterial infection whenever a diagnostic work up (blood cultures or tracheal aspirate culture) is ordered. The intervention strategy will consist of cytokine measurement from the sample and provision of a result based post-test probability within a few hours after sample collection. The null hypothesis states that the management arms will not differ with respect to antibiotic utilization rate, measured as the number of days on systemic antibiotic treatment per 1000 days of hospitalization. The secondary null hypothesis states that the arms will not differ with respect to costs associated with hospital acquired septic shock.

## Methods

### Design

The study is a multicentre randomized controlled trial comparing a new diagnostic treatment strategy for diagnosing bacterial infection versus standard care in critically ill newborns and children. During a 16-week period in 2003 we conducted a pilot study, which tested the intervention and data collection procedures, and led to modifications to the study design. The pilot study is outlined in detail below. In brief, physicians provide pre-test probabilities whenever they order a diagnostic work up for sepsis or ventilator-associated pneumonia (microbiological cultures). This includes any prescription of antibiotics. In the intervention arm, physicians are provided with cytokine results and the updated post-test probability. In the control arm no information is given.

### Eligibility criteria for participants

All patients admitted to the interdisciplinary neonatal or paediatric intensive care unit (ICU) of the Children's Hospital of Zurich are eligible. Patients who are referred to other wards within 24 hours after admission will be excluded from data analysis, because in these patients the decision to stop antibiotic treatment is no longer the responsibility of participating intensivists.

### Setting

The participating university hospital is the tertiary referral centre for Eastern and Southern Switzerland, and serves a population of approximately 3 million. The Department of Neonatology and Pediatric Intensive Care at the University Children's Hospital of Zurich contributes patients from its two ICUs, named unit A and unit B. Both units have average occupancy of 8–10 beds. The two units admit between 900 and 1000 patients annually, with the number of hospitalization days amounting to 5500 each year. The patient population in unit A includes infants of extremely low birth weight referred from other hospitals, critically ill children and adolescent patients, trauma victims and high-risk surgical patients. Unit B predominantly cares for infants and children who have undergone cardiac surgery.

### Intervention and controls

#### Control strategy

**Figure 1:**
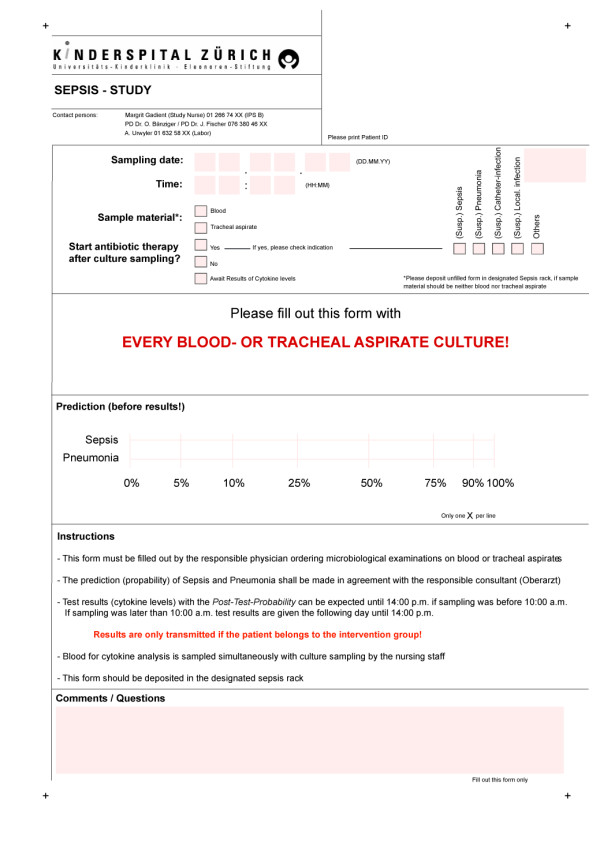
Order form for cytokine analysis. Physicians must enter date, time and material for microbiological examination. If antibiotic treatment is started or if a previously ordered treatment is changed, then the reason for this change must be checked in one of the boxes provided. Physicians must indicate their estimate of the likelihood of sepsis and ventilator associated pneumonia on the logarithmic visual-analogue scale. (The final form will be in German.)

For patients randomized to the control arm, if the physician orders microbial cultures then they are obliged to document their best estimate of the probability that the patient has sepsis or pneumonia on two logarithmic visual-analogue scales (range 0–100%). This documentation is mandatory and must be marked on the laboratory form (Fig. 1). In the control arm blood or tracheal aspirate specimens are not analyzed; thus, physicians do not receive any information beyond routinely available data. Under the control strategy antibiotic treatment is managed according to current recommendations (cessation of therapy after 48 hours provided that blood cultures remain negative).

#### Intervention strategy

**Figure 2:**
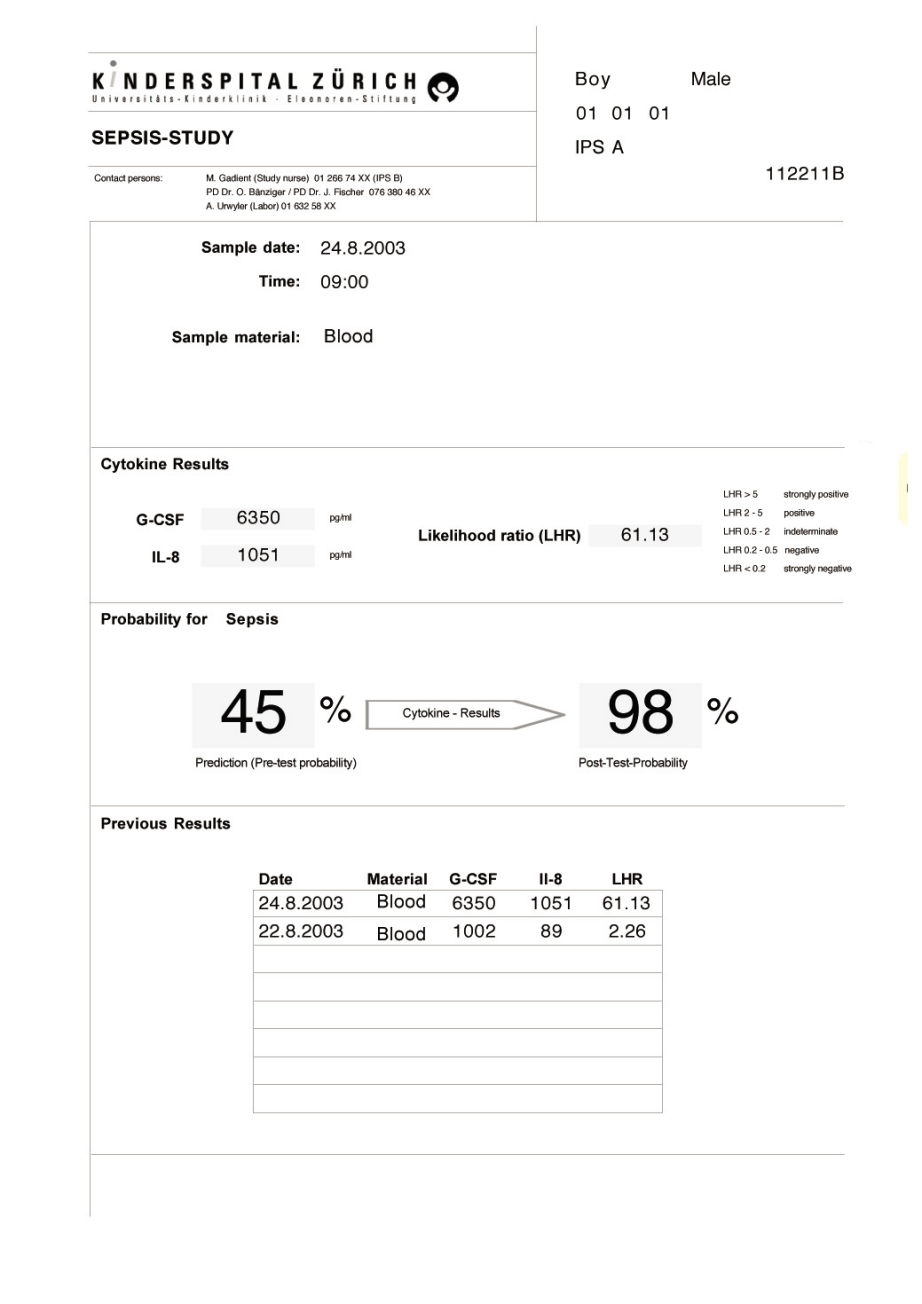
Result form. Results are presented in three ways: raw cytokine concentrations in pg/μl; cytokine concentration derived likelihood ratios for the presence of sepsis or pneumonia; and post-test probabilities of the presence of sepsis or pneumonia. G-CSF, granulocyte colony-stimulating factor; IL, interleukin. (The final form will be in German.)

If patients are randomized to the intervention arm, then physicians are also obliged to document their best estimate of the probability that the patient has sepsis or pneumonia, again on two logarithmic visual-analogue scales (range 0–100%). This documentation is again mandatory and must be marked on the laboratory form (Fig. 1). In the intervention arm, blood or tracheal aspirate specimens are analyzed and results are returned to the unit before 1 p.m. Physicians receive the raw cytokine values as well as the calculated likelihood ratio and the post-test probability (Fig. 2). This information is provided in addition to routinely available data. Provided that the available post-test probability indicates absence of infection, physicians are encouraged to stop antibiotic treatment. It is suggested that antimicrobial therapy be continued if the post-test probability indicates infection. It is important to note that the protocol provides only 'suggestions', and that the final decision regarding therapy is left to the discretion of the responsible clinician. This is similar to clinical routine, in which diagnostic results may suggest alterations to treatment decisions but they do not dictate treatment.

### Randomization

The units of randomization are calendar days. Randomization is generated through pre-specified assignment of 15 working days/month as intervention days. Physicians remain blinded to the allocation roster. Thirty minutes after the deadline for delivery of samples to the laboratory (10 a.m.), physicians are informed about the randomization status (control or intervention) of the day. In this way, physicians are able to adjust their decision making while they await test results if they so wish.

### Data collection

**Figure 3:**
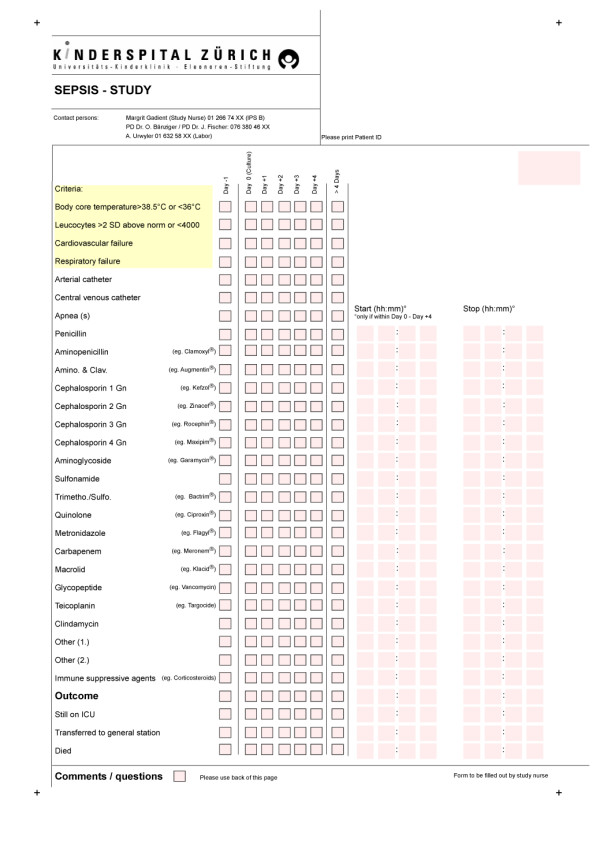
Clinical data record form. A trained study nurse collects all relevant clinical data for the day before and until 6 days after collection of blood and/or tracheal aspirate for microbiological examination. ICU, intensive care unit. (The final form will be in German.)

Routine sepsis work up includes collection of blood cultures, other microbial specimens where appropriate, and measurements of white blood cell count, including differential and plasma levels of C-reactive protein. Routine surveillance for ventilator-associated pneumonia comprises microbiological examination of the tracheal aspirate, including cultures. As described above, physicians must provide two probability estimates, one for the presence of sepsis and one for pneumonia, whenever they order a sepsis or pneumonia work up. This ensures that clinicians state their estimate before knowledge of the test result. These estimates (pre-test probabilities) are integrated with cytokine concentrations derived from likelihood ratios for sepsis or pneumonia using Bayes' theorem. The algorithms for calculating post-test probabilities are presented in Table 1. A study nurse records clinical data for both groups on the day preceding collection of culture specimens and on the following 6 days (Fig. 3). We will collect data on mortality, but this will not be included as a study outcome because of low mortality rates and the intended study size. Further data are collected from the hospital's database. This database contains all physician's reports, patient baseline data, routine laboratory results, pharmacology data, costs per patient and day of specific medications (e.g. fresh frozen plasma), and staff allocation.

### Cytokine measurement

Blood samples are collected until 10 a.m. in EDTA-containing vacutainers. Immediately thereafter they are centrifuged at 3000 rpm for 10 min and plasma removed for cytokine analysis. Tracheal aspirate samples are obtained through the endotracheal tube using a sterile suction system (Medinorm AG, Quierschied, Germany). Samples are centrifuged at 10,000 rpm for 5 min and cell free supernatant removed for analysis. Cytokine concentrations (tracheal aspirate and plasma) are simultaneously determined using fluorescent latex beads linked to monoclonal antibodies (R&D Systems, Abington, UK) marked after incubation and coupling with a second phycoerythrin monoclonal antibody (sandwich technique) (R&D Systems, Abington, UK). Final measurement and analysis is done on a Cytomics™ FC 500 Series analyzer (Beckman Coulter Inc., Fullerton, CA, USA).

### Pilot study

During a 16-week period in 2003, we conducted a pilot study in both paediatric ICUs. During this period, cytokine concentrations were available daily for all hospitalized patients so that the new laboratory marker could be implemented as part of routine diagnostic decision making. Clinical data were collected from all hospitalized patients for each day of their ICU stay by one of the investigators (TH). Several teaching sessions both for physicians and nurses were held to enhance the implementation process.

The pilot study revealed that the diagnostic test performance (combined likelihood ratio derived from plasma levels of IL-8 and G-CSF; receiver operating characteristic [ROC] 0.88) was similar to that of a published study (ROC 0.85) [9]. However, because clinicians were certain about the presence or absence of infection in half of the episodes, potentially clinically useful test results were found in fewer than a third of all episodes. Thus, we designed the randomized controlled trial as a test to rule-in or rule-out suspected infection only.

### Objectives and hypotheses

Our objectives are to achieve a clinically relevant reduction in overall antibiotic use and to reduce treatment costs caused by delayed diagnosis of nosocomial infection. In this study we will test the hypothesis that routine surveillance by determination of cytokine levels in plasma and tracheal aspirates will allow safe discontinuation of antibiotic therapy within 24 hours if the proposed laboratory prediction model indicates absence of infection. We regard a reduction in antibiotic exposure by 15% to be a clinically relevant effect. The second hypothesis we will test is whether early diagnosis reduces the morbidity and costs associated with hospital acquired infection. Ascertaining relevant indicators of morbidity and costs in all patients with culture proven bloodstream infection will operationalize this.

### Measures of outcome

The primary outcome measure is the rate of systemic antibiotic use per 1000 days of hospitalization (see details under Sample size calculation and statistical considerations). Secondary outcome measures are as follows (for all episodes of hospital acquired infection with positive blood cultures for the first 7 days following initiation of antibiotics after adjusting for important possible patient confounders): number of days free from mechanical ventilation (an indicator of respiratory failure); number of days free from inotropic support (an indicator of circulatory failure); costs for specific expensive medications (e.g. fresh frozen plasma); and nurse allocation (an indicator of treatment intensity).

### Sample size calculation and statistical considerations

At present, in the ICU antibiotic therapy is employed in 40% of patients, which represents a decline from our original survey conducted in 1998 (up to 80% of all patients) [3]. The expected reduction in antibiotic usage is 10–25%, with a clinically relevant reduction considered to be any reduction in excess of 10%. The minimum number of days of hospitalization in each arm required to detect a 10% reduction with a type I error under 5% and a power of 80% is 2300. The expected follow up rate is in excess of 90%. Because the unit of randomization is days and not individuals, an unknown intracluster (intraday) correlation coefficient must be considered. The standard χ^2 ^statistic, which assumes independence of individuals, may not be applicable. We may be forced to acknowledge the nested nature of the data (clustered randomized controlled trial) by using test statistics based on the generalized linear mixed model [12]. To safeguard against insufficient power we believe that the sample size must be increased to 25%, leading to a required accrual of 3000 hospitalization days per arm. Given the size of the participating units, this translates to a study duration of 24 months.

All analyses will be carried out on an intention-to-treat basis. This means that any antibiotic treatment course will be allocated according to the randomization status of the day on which the decision to withhold or to continue had to be made. This requires us to perform three subgroup analyses: antibiotic prescription prevalence according to the day's randomization status; antibiotic free days following the 4 days after any microbiological work up; and antibiotic free days during the week following any initiation of antibiotics.

### Stopping rules

Twelve months after initiating the trial, we will conduct an interim analysis at a two-sided *P *< 0.01 level. If the results indicate no trend toward a change (increase or reduction) in antibiotic treatment (curtailment from 48 to 24 hours) in prophylactic empirical therapy, and if there is no trend at the *P *< 0.1 level toward improved secondary outcomes, then the trial will be discontinued. The interim analysis implies that the result of the final analysis should be considered significant if *P *< 0.04.

## Discussion

A variety of publications report excellent diagnostic performance of new markers of infection [13,14]. However, a theoretically useful test may not necessarily provide clinically useful information. Most test accuracy studies derive their results from a subgroup of potentially eligible patients who satisfy unanimously accepted criteria for acceptance as cases or controls. Unfortunately, this practice suffers from the potential overestimation of the test accuracy [15] and, even more importantly, it disregards any clinical information that is available apart from that pertaining to the test under question.

In this randomized controlled trial we wish to assess the clinical efficacy of an innovative diagnostic procedure for the diagnosis of bacterial infection in newborns and critically ill children. It will evaluate whether this strategy results in a clinically relevant reduction in overall antibiotic usage, and whether the strategy is cost-effective by reducing treatment costs caused by delayed diagnosis of nosocomial infection.

One of the possible limitations of the study is the required extended study duration of 24 months. It is conceivable that experience gained from patients in the intervention arm or other factors attributable to the conduct of the study (for example increased awareness by physicians because of more conscious decision making) will also affect the control arm. This might lead to an altered prescription pattern in the control group, which would reduce our ability to find a significant difference between the study arms.

If the new test proves efficacious in clinical practice and is cost-effective, then it may become established as a routine marker of infection in this specific setting.

## Author's contributions

JF initiated the project and is the principal investigator. JF, TH, SH, DN and OB participated in the design of the study. JF and TH wrote the protocol. TH carried out the pilot study under supervision of JF. TH implemented the project into clinical routine. JF will carry out statistical analyses. All authors read and approved the final manuscript.

## Key messages

• 	Test accuracy should be evaluated prospectively with integrated bedside clinical information.

• 	The presented design of this ongoing RCT addresses these demands and shall test whether an innovative diagnostic procedure results in a relevant reduction in unnecessary antibiotic utilization and whether this new strategy proves to be cost effective.

## Competing interests

The author(s) declare that they have no competing interests.

## Abbreviations

G-CSF = granulocyte colony-stimulating factor; ICU = intensive care unit; IL = interleukin; ROC = receiver operating characteristic.

## Figures and Tables

**Table 1:** Equations for calculating post-test probabilities

Algorithm/parameters	Equations
Algorithm for sepsis	
Pre-test probability_s_	= prevalence of sepsis
Pre-test odds_s_	= pre-test probability_s_/(1 - pre-test probability_s_)
Likelihood ratio_s_	= exp(-8.2 + 0.85 × Ln [G-CSF_p_] + 0.7 × Ln [IL-8_p_])
Post-test odds_s_	= likelihood ratio_s _× pre-test odds_s_
Post-test probability_s_	= post-test odds_s_/(1 + post-test odds_s_)
Algorithm for pneumonia	
Pre-test probability_vap_	= prevalence of pneumonia
Pre-test odds_vap_	= pre-test probability_vap_/(1 - pre-test probability_vap_)
Likelihood ratio_vap_	= exp(-6.8 + 1.0 × Ln [G-CSF_t_])
Post-test odds_vap_	= likelihood ratio_vap _× pre-test odds_vap_
Post-test probability_vap_	= post-test odds_vap_/(1 + post-test odds_vap_)

The concentrations of granulocyte colony-stimulating factor (G-CSF) and interleukin (IL)-8 used in the above equations are in pg/μl. Definitions of subscript abbreviations: p, plasma; s, sepsis; t, tracheal aspirate; vap, ventilator associated pneumonia.
